# The Role of miRNAs during Endoplasmic Reticulum Stress Induced Apoptosis in Digestive Cancer

**DOI:** 10.7150/jca.62352

**Published:** 2021-09-23

**Authors:** Yujing Zhang, Shuai Huang, Gang Yang, Lianhong Zou, Xin Huang, Sulai Liu

**Affiliations:** 1 Key Laboratory of Molecular Epidemiology of Hunan Province, School of Medicine, Hunan Normal University, Changsha, 410081, China.; 2 Key Laboratory of Protein Chemistry and Developmental Biology of Fish of Ministry of Education, Hunan Normal University, Changsha, 410081, China.; 3 Hunan Provincial Institute of Emergency Medicine, Hunan Provincial People's Hospital/The First Affiliated Hospital of Hunan Normal University, Changsha, 410015, China.; 4 Department of Hepatobiliary Surgery, Hunan Provincial People's Hospital/The First Affiliated Hospital of Hunan Normal University, Changsha, 410015, China.

**Keywords:** ER stress, miRNAs, digestive cancers

## Abstract

Digestive cancer is one of the leading causes of cancer mortality in the world. Despite a number of studies being conducted, the exact mechanism for treating digestive cancer has not yet been fully understood. To survive, digestive cancer cells are subjected to various internal and external adverse factors, such as hypoxia, nutritional deficiencies or drug toxicity, resulting in accumulation of misfolded and unfolded protein in endoplasmic reticulum (ER) lumen further leading to ER stress and the unfolded protein response (UPR). During the last years, studies on the relationship between ER stress and microRNAs (miRNAs) has burst on the scene. miRNAs are non-coding RNAs with a length of 21~22nucleotides involved in post-transcriptional regulation of gene expression, which could be regarded as oncomiRs (tumor inducers) and tumor suppressors regulating cancer cell proliferation, invasion, and apoptosis by differently affecting the expression of genes related to cancer cell signaling. Therefore, investigating the interaction between ER stress and miRNAs is crucial for developing effective cancer treatment and prevention strategies. In this review, we mainly discuss miRNAs focusing on its regulation, role in ER stress induced apoptosis in Digestive cancer, expound the underlying mechanism, thus provides a theoretical foundation for finding new therapeutic targets of digestive cancer.

## Introduction

The endoplasmic reticulum (ER) is a peculiar sheets and tubules structure which is composed of a complex system of membranes that gives rise to the nuclear envelope (NE) and peripheral ER 1. The shape and architecture of ER determine the function of it. The ER is responsible for the synthesis, folding, as well as post-translation modification of secreted and membrane proteins 2, moreover, it is a container for storing calcium ions. ER is a dynamic organelle which can quickly adapt in order to satisfy various cellular requirements in response to physiological or pathological stimuli 3. The ER adapts to stress by activating a signaling cascade known as the ER stress response 4. As guardians of the ER, a three-branch system composed by three transmembrane proteins will be activated during ER stress response. This system is called unfolded protein response (UPR), which undergoes activation upon accumulation of misfolded proteins or unfolded proteins as well as excess release of calcium on account of leakage of the membranes 2. The three branches are composed of protein kinase R (PKR)-like ER kinase (PERK), inositol-requiring enzyme 1 (IRE1) and activating transcription factor 6 (ATF6), orchestrate the major regulatory circuits to ensure ER homeostasis 5. A growing number of studies indicate that ER stress involves numerous diseases including but not limited to neurodegenerative disease 6 like Alzheimer's disease 7, Chronic Obstructive Pulmonary Disease and Idiopathic Pulmonary Fibrosis 8, disease of immune system 2, diabetes 9, cardiovascular disease 10, as well as various cancer. Digestive cancer is one of the common malignant tumors with very poor overall survival worldwide, including esophageal cancer, gastric cancer, colorectal cancer, pancreatic cancer and liver cancer. Similar to other cancers, digestive cancers cells experiencing hypoxia undergo metabolic alterations accompanying with ER stress. For example, Aloe-Emodin could induce ER stress mediated apoptosis via upregulation of C/EBP homologous protein (CHOP) and caspase-12 expression in colorectal cancer cells 11.

The relationship between ER stress and microRNAs (miRNAs) is a hot topic in the field of medical research. miRNAs are 21-22nt small non-coding RNAs (ncRNAs) highly conserved among species that modulate gene expression, which mainly via recognition of cognate sequences and interference of transcriptional, translational or epigenetic processes 12-14. During the last decades, a major discovery in biology was the discovery of miRNAs, which provides new insight on the post-transcriptional regulation of gene expression and cancer research. Human miRNA genes are frequently located at fragile sites and genomic regions involved in cancers, suggesting that miRNAs may play a key role in in the pathogenesis of human cancers. It has been reported that miRNAs could be regarded as valuable instruments in tumor diagnosis and the prognosis of digestive cancers (affecting the esophagus, stomach, intestine, colorectum, liver and pancreas) 15. miR‑373 exerts anti‑tumor functions in human liver cancer by targeting Rab22a 16 and overexpression of miR-17 is correlated with liver metastasis in colorectal cancer 17, both confirming that miRNAs play a significant part in digestive cancers.

In consideration of both miRNAs and ER stress have important effects on the genesis as well as development of digestive cancer, here, we discussed the accelerative roles of miRNAs during the ER stress, elucidating the underlying mechanism thus providing a theoretical basis for considering interaction of miRNAs and ER stress as a potential therapeutic strategy ford digestive cancer.

## The relationship between ER stress-induced apoptosis and digestive cancer

In recent years, many studies have found that ER stress is closely related to a variety of cancers, including cancers of the digestive system. Three key factors of ER stress-related pathways, PERK, IRE1 and ATF6, activate corresponding signaling pathways and unfolding protein responses when separated from ER stress molecular chaperone glucose regulatory protein 78 (GRP78) 18. These three paths are both relatively independent and interrelated. Under ER stress, the activation of these three pathways enhanced the ability of protein folding, thus maximizing the adaptability and survival ability of cells to environmental changes, but too long or too intense ER stress would induce cell apoptosis 19. These three key factors and their related signaling pathways play a critical role in the regulation of the occurrence and development of cancer in the digestive system.

### PERK

PERK is an important ER transmembrane protein that participates in UPR by reducing protein translation and regulating oxidative stress^20^. Oligomerization and autophosphorylation of PERK stimulates eukaryotic translation initiation factor 2α (eIF2α) phosphorylation in response to stimulation of misfolded or unfolded proteins. Subsequently, phosphorylated eIF2α promotes transcription of activate transcription factor 4 (ATF4), which aggregates on the promoters of target genes, including transcription factor CHOP, growth arrest and DNA damage inducible protein 34 (GADD34), and activate transcription factor 3 (ATF3), facilitating translation of downstream target genes 21. PERK pathway can induce apoptosis of cancer cells under certain conditions, which is the key mechanism for many cancers therapy drugs to exert therapeutic effect. Cinchonine, a natural compound with anticancer activity, can promote the phosphorylation of PERK and eIF2α in a variety of hepatocellular carcinoma (HCC) cells, and significantly increase the protein level of CHOP, thus inducing the apoptosis of HCC cells 22. In gastric cancer, the activation of PERK significantly increases the G-1-induced apoptosis, while the silencing of PERK leads to increased cell apoptosis, suggesting that the PERK pathway may enhance the therapeutic effect of anti-cancer drugs 23. In addition, the PERK activator CCT020312 can induce ER stress and significantly inhibit the proliferation ability of colorectal cancer cells, which also further improve the sensitivity of colorectal cancer cells to chemotherapy drugs by the activated PERK 24. Similarly, Anlotinib (AL3818), a drug that has been proved to have anti-tumor activity recently, has an anti-proliferation effect on pancreatic cancer cells related to PERK by increase the accumulation of reactive oxygen species in cancer cells activating of the PERK/eIF2α/ATF4 pathway 25.

### IRE1

IRE1 is a transmembrane protein located in the ER and consists of two functional domains, including a C-terminal cytoplasmic effector domain and an N-terminal lumen sensor domain 26. IRE1 has two functions: protein kinase and endoribonuclease. The oligomeric and autophosphorylation of IRE1α can activate the endonuclease activity of either unfolded or misfolded proteins 27. After activation, the substrate precursor X-box binding protein 1 (XBP-1) and mRNA intron are spliced by IRE1α to produce mature active XBP-1 protein 28. XBP-1 binds to downstream gene promotors involved in UPR and ER associated degradation (ERAD), thereby regulating their expression and restoring ER homeostasis 29. Therefore, XBP-1 can increase the expression level of CHOP. Apoptosis-signaling kinase-1 (ASK-1) activates apoptosis by IRE1α. ASK-1 activates Jun-N-terminal kinase (JNK) and p38 mitogen-activated protein kinase (p38MAPK), leading to apoptotic cell death 30. IRE1 pathway is a key pro-survival/pro-apoptotic pathway in ER stress. It has shown that the activation of IRE1-XBP1 signaling inhibits the expression of desmoplakin enhancing the migration ability of liver cancer cells 31. IRE1 may also play a tumor suppressive role in cancers of the digestive system. Kaempferol, a flavonoid compound with strong anticancer effects, also induce gastric cancer cell death by activating IRE1-JNK-CHOP pathway 32. However, the activation of IRE1-XBP-1 may enhance the migration ability of colorectal cancer cells and thus promote the progression of colorectal cancer 33. As for pancreatic cancer, the IRE1α-XBP1 signaling pathway has previously been shown to contribute to pancreatic cancer cell invasion in xenograft models 34. These results suggest that IRE1α may also be an effective treatment target for pancreatic cancer.

### ATF6

ATF6 is a transmembrane protein associated with ER stress that is different from other ER stress factors and is the least well understood. Under ER stress, ATF6 translocate to Golgi apparatus. In the Gorky compartment, ATF6 is cleaved into an activated form called short ATF6 (sATF6) 35. After activation, sATF6 translocate to the nucleus and interacts with ATF/cAMP response elements and ER stress related elements in homologous or heterodimer form 36. In the initial stage of ER stress, ATF6 promotes the increase of ER capacity and ER protein folding ability through target genes, thus restoring ER homeostasis. On the other hand, ATF6 promotes apoptosis through CHOP when ER stress is prolonged 37. Sorafenib (Sor), a clinical standard therapy for advanced HCC, significantly increases the intensity of ATF6 immunofluorescence labeled in HCC cells, accompanying with the up-regulated expression of ATF6 protein, suggesting ATF6 pathway may be one of the mechanisms of SOR 38. Dehydroeffusol (DHE) shows the anti-cancer effects on gastric cancer by increased the expression of ATF6 39. Moreover, activated ATF6 could induce intestinal dysregulation and innate immune response and promote the occurrence of colorectal cancer 40. In connection with this, studies have shown that the expression of colon cancer oncogene CIP2A is positively correlated with the expression of ATF6, and ATF6, as a transcription factor, directly bind to the CIP2A promoter in turn. Similarly, immunohistochemical analysis of tissue microarray from a cohort of colon cancer patients showed that high expression levels of ATF6 were associated with a tendency for poor prognosis 41. These results suggest that ATF6 may be highly expressed in colorectal cancer and mainly plays a role in promoting the survival of cancer cells. In pancreatic cancer, however, ATF6 may plays a pro-apoptotic role. Studies have shown that in pancreatic cancer BXPC -3 cells, the upregulated ATF6 levels is closely related to cell apoptosis induced by Tanshinone (TAN)-IIA 42.

## miRNAs and digestive cancer

miRNAs are abnormally expressed in many cancers of the digestive system, which modulate the expression of cancer-related genes by directly targeting. Importantly, because of the abnormal expression of miRNAs in cancer, it is also a promising biomarker and target for cancer diagnosis and treatment. Here, the miRNAs mentioned in the review that are associated with cancers of the digestive system are listed in Table 1.

The abnormally low expression of miR-101 in a variety of digestive cancers including liver, gastric, colorectal and pancreatic cancer, playing a critical role in cancer suppression. In liver cancer, miR-101 targets nemo-like kinase (NLK) and plays an inhibitory role by inhibiting NLK activity 43. In gastric cancer, the ectopic expression of miR-101 significantly inhibits cell proliferation, migration and invasion by targeting enhancer of zeste homolog 2 (EZH2), Cyclooxygenase-2 (COX-2), Myeloid cell leukemia-1 (MCL-1) and Fos. In addition, animal studies have suggested that miR-101 may also inhibit tumor growth *in vivo* 44. In colorectal cancer, miR-101 significantly down-regulated the expression of sphingosine kinases 1 (SPHK1) mRNA and protein at the molecular level, leading to the production of pro-apoptotic ceramides in the above colorectal cancer cells, thereby inhibiting the growth of colorectal cancer cells. In addition, miR-101 enhanced paclitaxel-induced anti-HCT-116 activity *in vivo* 45.

amiR-132 is also abnormal expressed in digestive cancers. The transfection of miR-132 in liver cancer cells significantly inhibits cell proliferation by promoteing pro-apoptotic genes and inhibiting pro-survival genes, playing an inhibitory role in liver cancer cells^46^. Upregulated miR-132 in gastric cancer inhibits the translation process by binding to the 3'-untranslated region (3' -UTR) of Forkhead box O class protein 1 (FoxO1) messenger RNA (mRNA) to inhibit the inhibitory effect of FoxO1 on cells and promote the development of gastric cancer 47. In contrast, the expression of miR-132 is decreased in colorectal cancer, exerting a tumor suppressive effect. miR-132 was associated with tumor size, distant metastasis, and tumor node metastasis (TNM) stage in colorectal cancer. The ectopic expression of miR-132 could significantly inhibit the invasion ability and epithelial mesenchymal transformation of colorectal cancer cells, which may be due to its target ZEB2, the regulatory factor regulating epithelial mesenchymal transformation 48.

miR-145 plays an inhibitory role in liver cancer by targeting histone deacetylase 2 (HDAC2) 49. In gastric cancer, miR-145 directly targets transcription factor specificity protein 1SP1 to down-regulate the expression of matrix metalloproteinase-9 (MMP-9) and Cyclin D1, inhibiting cell growth and invasion 50. Overexpression of miR-145 in colorectal cancer inhibits cell proliferation, motility, and invasion *in vitro*, as well as tumor growth and lung metastasis. This antitumor effect may be due to its direct interaction with the 3'-UTR of Fascin-1 messenger RNA (mRNA), thus down-regulate the mRNA and protein expression levels 51.

miR-148b significantly inhibits tumorigenicity *in vivo*, possibly due to its target neuropilin-1 (NRP1), which is involved in tumor initiation, metastasis and angiogenesis 52. miR-148b is correlated with tumor size in gastric cancer, and could inhibit the proliferation of gastric cancer cells and tumorigenicity *in vivo* by targeting cholecystokinin-B receptor (CCKBR) 53. Overexpression of miR-148b in colorectal cancer cells inhibits cell proliferation *in vitro* and tumorigenicity *in vivo* by targeting the cholecystokinin-2 receptor (CCK2R) gene 54.

miR-21 promoting liver cancer cells growth and proliferation by targeting Sprouty1 55. In gastric cancer, miR-21 expression is correlated with the degree of tumor differentiation, local invasion and lymph node metastasis by targeting the tumor suppressor Phosphatase and Tensin Homolog deleted on Chromosome 10 (PTEN) 56. In colorectal cancer, patients with poor differentiation, lymph node metastasis and advanced TNM have significantly high expression of miR-21, which targets PTEN at the post-transcriptional level to regulate the proliferation and invasion of colorectal cancer cells. Inhibition of miR-21 expression in colorectal cancer cells can reduce cell proliferation, migration and invasion, induce cell apoptosis, and inhibit cell cycle progression 57.

In liver cancer, miR-214 is low expressed and plays a tumor suppressor role, which is related to ER stress related factors 58. In a variety of gastric cancer cell lines, miR-214 is highly expressed and targets the tumor suppressor PTEN, playing a cancer-promoting role 59. miR-214 is down-regulated in colorectal cancer and profoundly associated with lymphatic metastasis. *In vitro* and *in vivo* experiments showed that miR-214 mediated the inhibiting the function of Forkhead box D3 (FOXD3) on proliferation, invasion and metastasis by targeting mediator complex subunit 19 (Med19) 60.

Inhibition of miR-221 expression in liver cancer cells could reduce cell proliferation, clonability, migration/invasion ability, and induce G1 arrest or apoptosis by its targets, including BMF, BBC3, and AngPTL2. In addition, silencing miR-221 significantly inhibited the growth of HCC xenograft in nude mice 61. In gastric cancer, high expression of miR-221 is significantly associated with advanced lymph node metastasis, local invasion, and lymphatic metastasis. Moreover, overexpression of miR-221 is an adverse prognostic factor for overall survival in patients with gastric cancer 62. The high expression of miR-221 contributes to the metastasis of colorectal cancer, which also regulates the migration and invasion of colorectal cancer by targeting reversion inducing cysteine rich protein with kazal motifs (RECK) 63.

In liver cancer, miR-143 targets HCC suppressor Oxysterol binding related proteins 8 (ORP8), which can inhibit the development of liver cancer by inducing FASL through ER stress response 64. Both miR-143-5p and miR-143-3p are significantly down-regulated in various gastric cancer cell lines and shows anti-gastric cancer effects evidenced by inhibiting the activity of COX-2 65.

miR-103a-3p is highly expressed and associated with poor prognosis of gastric cancer, which also increases the proliferation of gastric cancer cells by targeting activating transcription factor 7 (ATF7) 66. In colorectal cancer, miR-103A-3p is also highly expressed and targets Pyruvate dehydrogenase E1 component subunit Beta (PDHB) to inhibit the development of colorectal cancer *in vitro* by affecting proliferation, invasion and ER stress 67.

## miRNAs regulate the function of ER stress in digestive cancer

As mentioned above, both ER stress and miRNAs play important regulatory roles in the development and progression of cancers of the digestive system. Functionally, they have many similarities and are closely related. For example, ER stress and miRNAs both play a role in cancer inhibition and promotion. Moreover, under certain conditions, miRNAs can regulate the effects of ER stress on digestive cancer, so as to play a regulatory role in the occurrence and development of digestive cancer. Here, miRNAs that regulate ER stress function in digestive cancer are listed in Table 2.

The down-regulated of miR-199a/214 induce apoptosis of liver cancer cells. Meanwhile, XBP-1 has been found to be the target of miR199a/214, suggesting that miR199a/214 plays an inhibitory role in liver cancer by inhibiting XBP-1-related pro-survival pathways 58. On the contrary, miR-3091-3p promotes apoptosis of liver cancer cells through pro-apoptotic pathways of ER stress, because miR-3091-3p inhibits autophagy and enhances ER stress-induced cell apoptosis via directly targeting autophagy-related protein 9b (Atg9b) 68. The evidence indicates that the regulation of miRNA on the function of ER stress is not limited to direct interaction, but also indirectly regulate ER stress via its target genes. For example, miR-663 play a key regulatory roles in of ER stress-mediated apoptosis resistance of liver cancer cells by targeting transforming growth factor beta 1 (TGFB1) 69. Likewise, miR221/222 are involved in the regulation of ER stress-induced apoptosis. Interestingly, this process is also involved in p27(KIP1)- and MEK/ERK-regulated cell cycle progression, suggesting that the regulation between miRNA and ER stress may involve other mechanisms related to the regulation of cancer cell life process 70. Enhanced pro-apoptotic and anti-proliferative properties of Tacrolimus are associated with PERK pathway, while it inhibits the expression of miR-92A-1-5p, miR-197-3p, miR-483-3p and miR-720 in HepG2 cells. These results suggest that the abnormally expressed miRNAs may be involved in regulating ER stress-induced apoptosis in liver cancer 71.

In gastric cancer, loss of miR-370 promotes ER stress-induced apoptosis and inhibit the proliferation of cancer cells 72. In this case, miR-370 also does not directly target ER stress related factors, but instead regulates ER stress by targeting HERPUD. miR-379-5p inhibits the expression of GRP78 via directly targeting. GRP78 expression is negatively correlated with the growth inhibition rate of DDP (cisplatin) resistant gastric cancer cells, and functional analysis showed that enhanced miR-379-5p expression inhibits the proliferation of DDP resistant gastric cancer cells 73. This may be due to the fact that miR-379-5p inhibits the pro-survival pathway of ER stress in gastric cancer cells after acting on GRP78, leading to apoptosis. In addition, miR-143 is functionally antagonistic with ER stress in gastric cancer. In short, ORP8, the target of miR-143, activates NF-**κ**B by ER stress to induce apoptosis via Fas/FasL pathway. In this process, miR-143 indirectly and negatively regulated ER stress 64. miR-133a-3p also increase Bufothionine-induced apoptosis of gastric cancer cells by regulating ER stress 74.

In colorectal cancer, miR-451 inhibits colorectal cancer cell proliferation and induces apoptosis by targeting the ER carrier protein B-cell receptor-associated protein 31 (BAP31). Then, Bax, PERK/eIF2α/ATF4/CHOP and other ER stress apoptotic pathway proteins be further activated, resulting in the apoptosis of colorectal cancer cells 75. miR-7112-3p directly targets PERK and activates PERK/ATF4/CHOP/Caspase-level pathway to attenuate apoptosis of colorectal cancer CX-1 cells treated with DVDMS-PDT 76. miR-103a-3p, an up-regulated miRNA in colorectal cancer, inhibits the pro-apoptosis function of ER stress 67. miRNA and mRNA regulatory networks indicate that some miRNAs in HCT116 cells respond to folate deficiency closely related to the progression of colorectal cancer by regulating the expression of genes related to ER stress. Additionally, folate deficiency may cause some miRNAs (miR-379-5p, miR-218-5p, miR-1-3p, miR-486-3p, miR-24-3p, and miR-3182-3p, miR-132-3p, miR-483-3p, and miR-30d-5p) upregulated to inhibit the expression of key genes in the ER stress 77, suggesting that the regulation between these miRNA and ER stress pathway genes may be one of the mechanisms that regulate the progression of colorectal cancer.

To sum up, miRNAs and ER stress can not only independently regulate the digestive system cancer, but also co-determine the fate of digestive cancer cells by regulating the function of ER stress. Moreover, ER stress function can be directly modulated by miRNAs in digestive system cancers, that is, miRNAs can directly target ER stress-related factors. ER stress function can also be indirectly regulated by miRNAs, and other signaling pathways of digestive cancer are also involved in this process. The indirect regulation between the two may be a complex multiphase regulation network. To investigate the regulatory role of miRNAs and ER stress and perform intervention is of great significance for the formulation of effective treatment and prevention strategies for digestive cancer.

## Conclusion

Digestive cancer is one of the main causes of death worldwide. Although a number of research on its mechanism and therapy have been conducted, the effective therapeutic target for treating digestive cancer has not yet been fully understood. Hostile microenvironmental conditions of digestive cancer cell, including hypoxia, bad nutrients and oxidative stress induce cellular stress such as ER stress and trigger UPR to maintain cellular homeostasis. However, persistent ER stress triggers the apoptotic pathway. The effects of ER stress on digestive cancer cells are both positive and negative. Therefore, blocking the adaptive pathway of ER stress or promoting the apoptotic pathway of ER stress may be an effective anti-cancer strategy. Under ER stress, digestive cance cells undergo a series of biological changes to survive, including miRNAs expression. miRNAs directly or indirectly influence UPR signaling to determine cell fate. Emerging evidence suggests that miRNAs play important roles in the development of digestive cancer (Table 2). Some miRNAs may target oncogenes and/or tumor suppressors closely related to digestive cancer, while others may be directly control differentiation and apoptosis of digestive. Understanding of the function of miRNAs especially its regulation on ER stress induced apoptosis in digestive cancer is providing the new insights on the molecular basis of digestive cancers, and new biomarkers for cancer diagnoses and cancer therapy.

## Figures and Tables

**Figure 1:**
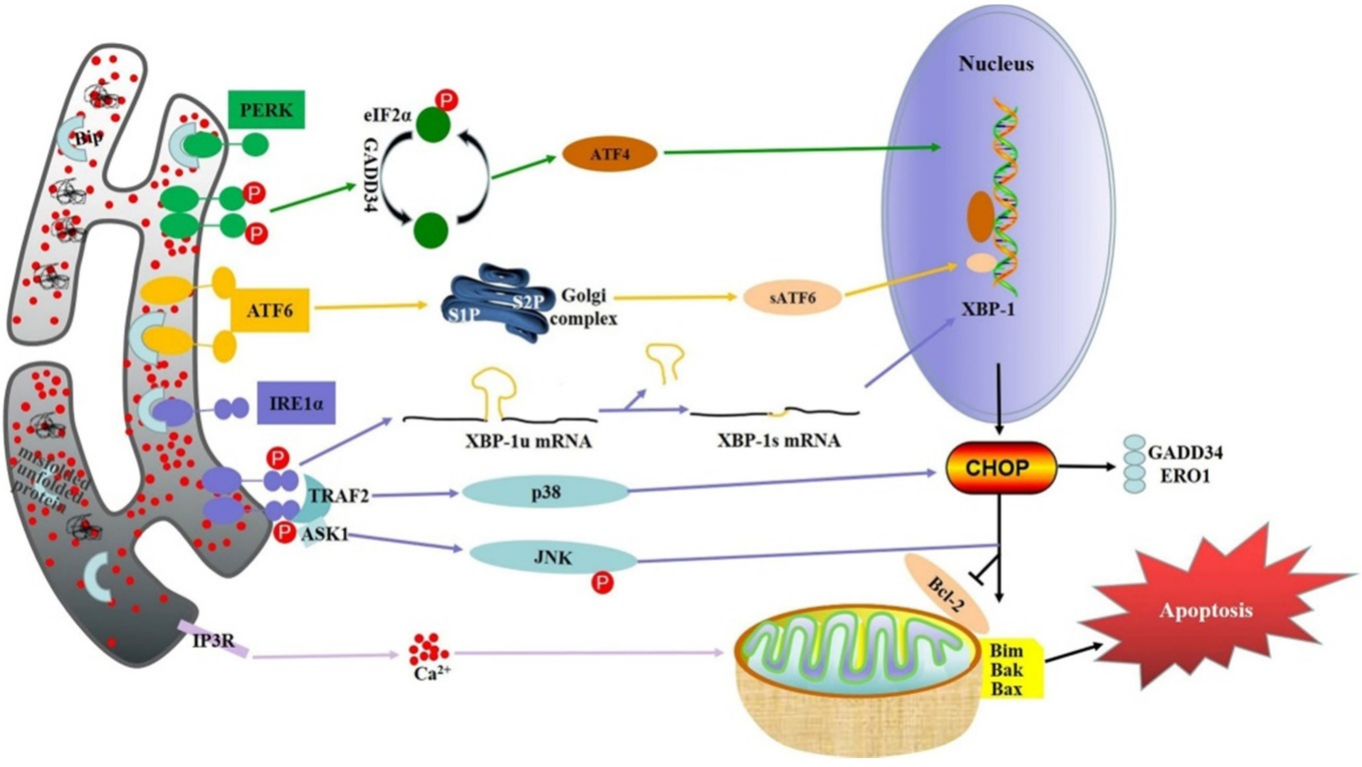
ER stress-induced apoptosis in digestive cancer.

**Figure 2:**
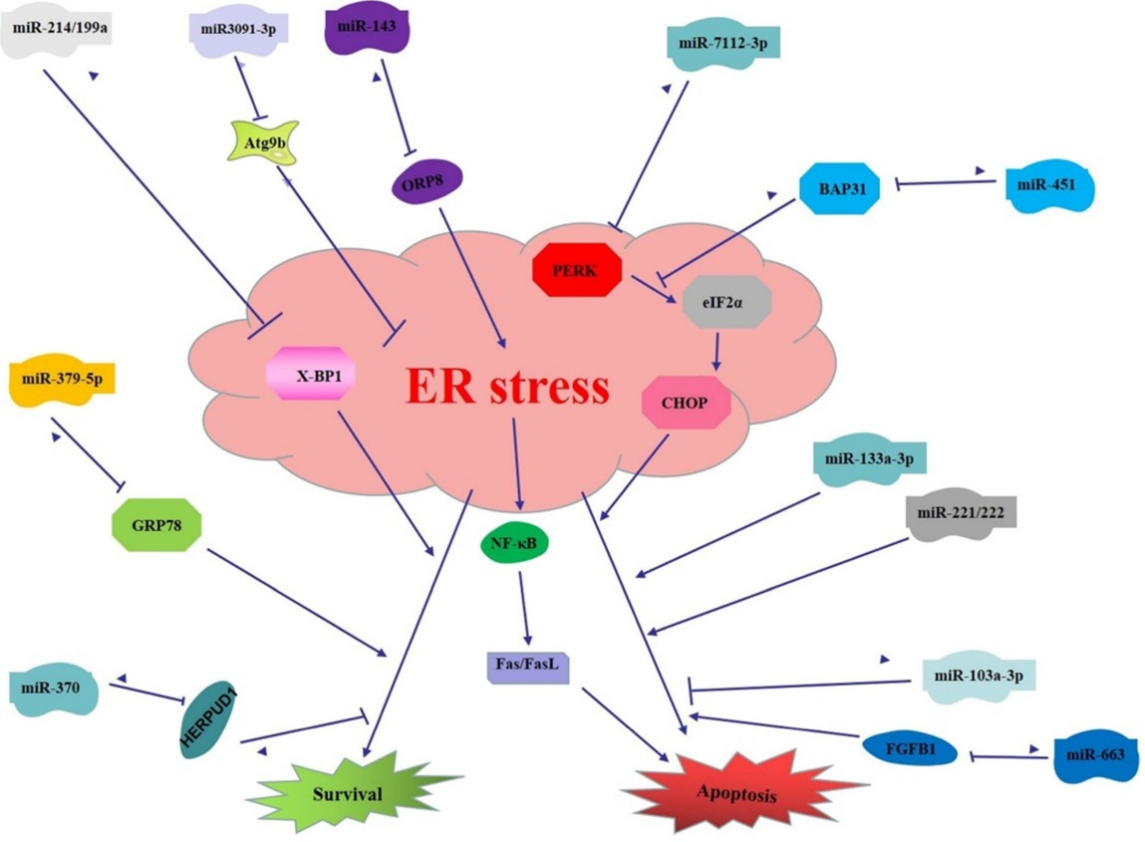
** miRNAs regulate the function of ER stress in digestive cancer.** (1) miR199a/214 targets XBP-1 to induce apoptosis in liver cancer; miR-3091-3p targets Atg9b to enhance ER stress induced apoptosis in liver cancer; miR-663 targets FGFB1 to inhibit ER stress induced apoptosis in liver cancer; miR221/222 promotes ER stress induced apoptosis in liver cancer; (2) miR370 targets HERPUD1 to promote survival pathway of ER stress in gastric cancer; miR-379-5p targets GRP78 to inhibit survival pathway of ER stress in gastric cancer; miR-143 targets ORP8 to inhibit ER stress and downstream pathway to inhibit apoptosis in gastric cancer; miR-133a-3p enhances ER stress induced apoptosis in gastric cancer; (3) miR-451 targets BAP31 thus inhibit ER stress induced apoptosis in colorectal cancer; miR-7112-3p targets PERK to inhibit ER stress induced apoptosis in colorectal cancer; miR-103a-3p inhibits pro-apoptosis function of ER stress.

**Table 1:** miRNAs abnormally expressed in caners of digestive system

miR ID	Type of cancer	Expression	Targets	References
miR-101	liver cancer	down	Nemo-like kinase (NLK)	43
	gastric cancer	down	EZH2, Cox-2, Mcl-1, Fos	44
	colorectal cancer	down	sphingosine kinase 1 (SphK1)	45
miR-132	liver cancer	down	p-AKT, Survivin	46
	gastric cancer	up	FoxO1	47
	colorectal cancer	down	ZEB2	48
miR-145	liver cancer	down	HDAC2	49
	gastric cancer	down	SP1	50
	colorectal cancer	down	fasin -1	51
miR-148b	liver cancer	down	NRP1	52
	gastric cancer	down	CCKBR	53
	colorectal cancer	down	CCK2R	54
miR-21	liver cancer	up	Sprouty1	55
	gastric cancer	up	PTEN	56
	colorectal cancer	up	PTEN	57
miR-214	liver cancer	down	X-BP1/NF-κB	58
	gastric cancer	up	PTEN	59
	colorectal cancer	down	MED19	60
miR-221	liver cancer	up	BMF, BBC3, ANGPTL2	61
	gastric cancer	up	-	62
	colorectal cancer	up	RECK	63
miR-143	liver cancer	up	ORP8	64
	gastric cancer	down	COX-2	65
miR-103a-3p	gastric cancer	up	ATF7	66
	colorectal cancer	up	MEG3, PDHB	67

**Table 2:** miRNAs interplay to ER stress in digestive cancers

Cancer Type	miRNA	Expression in cancer	Related Genes/Proteins/Pathways	Relationship with ER stress	References
Liver Cancer	miR199a/214	down	X-BP1/NF-κB	Inhibiting ER stress pro-survival function	58
	miR-3091-3p	down	ATg9b, p62, LC3	Enhancing ER stress pro-apoptosis function	68
	miR-663	up	TGFB1	Inhibiting ER stress pro-apoptosis function	69
	miR221/222	down	p27 (Kip1), MEK/ERK	Enhancing ER stress pro-apoptosis function	70
	miR23a-3p	up	PTEN/PI3K/AKT, PDL-1	Induced by ER stress	78
	miR-92a-1-5p/miR-197-3p/miR-483-3p/miR-720	/	PERK, p53, p21, FKBP12, FKBP51	Inversely related to ER stress in function	71
	miR-22-3p/miR-376a-3p/miR-663b/miR-886-5p/ miR-1300/ miR-1303	/	PERK, p53, p21, FKBP12, FKBP51	Positively related to ER stress in function	71
	miR-143	up	ORP8, Fas/FasL	Functionally opposite with ER stress	64
Gastric Cancer	miR370	up	circ_002117, HERPUD1	Inhibiting ER stress pro-apoptosis function	72
	miR379-5p	down	LINC00665, GRP78	Inhibiting ER stress pro-survival function	73
	miR-133a-3p	down	PI3K/Akt, IGF1R	Enhancing ER stress pro-apoptosis function	74
Colorectal Cancer	miR-451	down	BAP31	Enhancing ER stress pro-apoptosis function	75
	miR-7112-3p	up	PERK/ATF4/CHOP	Inhibiting ER stress pro-apoptosis function	76
	miR-379-5p/miR-218-5p/miR-1-3p/miR-486-3p/miR-24-3p/mir -3182-3p/miR-132-3p/miR-483-3p/miR-30d-5p	/	ER stress related pathway	Inhibiting expression of critical ER stress related genes	77
	miR-103a-3p	up	MEG3, PDHB	Inhibiting ER stress pro-apoptosis function	67
